# Genome-wide SNPs in the spiny lobster *Panulirus homarus* reveal a hybrid origin for its subspecies

**DOI:** 10.1186/s12864-022-08984-w

**Published:** 2022-11-12

**Authors:** Ahmad Farhadi, Andrew G. Jeffs, Shane D. Lavery

**Affiliations:** 1 Department of Natural Resources and Environmental Engineering, Shiraz University, Shiraz, Iran; 2 School of Biological Sciences, The University of Auckland, Auckland, New Zealand

**Keywords:** Genome-wide SNPs, *Panulirus homarus* complex, Admixture, Demography

## Abstract

**Background:**

Evolutionary divergence and speciation often occur at a slower rate in the marine realm due to the higher potential for long-distance reproductive interaction through larval dispersal. One common evolutionary pattern in the Indo-Pacific, is divergence of populations and species at the peripheries of widely-distributed organisms. However, the evolutionary and demographic histories of such divergence are yet to be well understood. Here we address these issues by coupling genome-wide SNP data with mitochondrial DNA sequences to test the patterns of genetic divergence and possible secondary contact among geographically distant populations of the highly valuable spiny lobster *Panulirus homarus* species complex, distributed widely through the Indo-Pacific, from South Africa to the Marquesas Islands.

**Result:**

After stringent filtering, 2020 SNPs were used for population genetic and demographic analyses, revealing strong regional structure (F_ST_ = 0.148, *P* < 0001), superficially in accordance with previous analyses. However, detailed demographic analyses supported a much more complex evolutionary history of these populations, including a hybrid origin of a North-West Indian Ocean (NWIO) population, which has previously been discriminated morphologically, but not genetically. The best-supported demographic models suggested that the current genetic relationships among populations were due to a complex series of past divergences followed by asymmetric migration in more recent times.

**Conclusion:**

Overall, this study suggests that alternating periods of marine divergence and gene flow have driven the current genetic patterns observed in this lobster and may help explain the observed wider patterns of marine species diversity in the Indo-Pacific.

**Supplementary Information:**

The online version contains supplementary material available at 10.1186/s12864-022-08984-w.

## Background

In comparison to the terrestrial environment, historical population divergence and speciation in the marine environment is believed to have occurred at a relatively slow pace, with historical interactions poorly understood, due to difficulties in easily observing the most important barriers to gene flow in this realm. Past heterogeneous historical changes in the marine environment and currents, have affected connectivity and dispersal patterns over time, resulting in complex patterns of speciation that are not easily discerned from the present. A variety of speciation processes have been proposed to explain patterns of marine diversity in the Indo-Pacific, including speciation from a center of origin in the Indo-Australian Archipelago [1], vicariant speciation between the Indian and Pacific Oceans (across the “marine Wallace’s Line” [2]), and peripheral (centripetal) speciation of isolated populations [3]. However, most of the clear genetic evidence of marine speciation processes in this region are taken from either long-diverged species [4], or separate populations within a species that are likely to be a long way from speciation e.g. [5];. As such, these examples are often contradictory in their conclusions as to the major forces driving speciation. Therefore, examining a species complex with a wide spatial and temporal scale of distribution with geographically overlapping lineages, and with a divergence dynamic evidenced through reproductive isolation can help us to understand the processes of marine speciation.

Examining situations where there are historical patterns of lineage divergence and secondary contact in both core and peripheral populations, are important not only in understanding speciation process, but also for the current conservation and management of species in the marine environment. Spiny lobsters (i.e., Palinuridae) with wide distributions and high species diversity can be viewed as an excellent model for understanding demographic patterns of marine diversity and speciation. Furthermore, their extended pelagic larval periods (4–12 months [6];) could be expected to facilitate wide dispersal and high levels of population connectivity. However, previous studies on several species of spiny lobsters (using mitochondrial DNA (mtDNA) and nuclear microsatellites) have revealed a surprising degree of genetic structure and divergent lineages, especially at the peripheries of the distributions of some species [7–11]. However, the demographic complexities of those relationships have so far eluded discovery. While it is clear that both genetic divergences and genetic mixing have occurred in the evolutionary past of these species, the complexity of the demographic patterns and timing of these events have not been adequately resolved.

In marine organisms, genome-wide SNP discovery has been of considerable assistance in improving our understanding of the degree of population genetic differences [12, 13], detection of secondary contact [14], and the effect of local environmental adaptation [15]. The joint allele frequency spectrum (AFS) from genome-wide SNP data has recently been used successfully to investigate historical demography of several non-model marine species (e.g., [16]). Understanding the degree of secondary contact, admixture and the potential fitness of hybrids or parental populations can also be useful for aquaculture, through an understanding of the genetic diversity within target species, and by helping to identify the source of unique broodstock.

Spiny lobsters play a key ecological role as predators in marine ecosystems in many parts of the world. They are also a major economic resource for artisanal and commercial fisheries in tropical, subtropical and temperate ocean regions of the world, with combined annual landings valued at over US$1 billion [17]. Because of their high economic value and increasing demand in markets, their populations are frequently stressed through overfishing [18] in addition to potential widespread habitat destruction and decreases in larval recruitment driven by climate change processes [19, 20]. Despite the global significance of spiny lobsters, their phylogeographic patterns and adaptive divergence are poorly understood.

Among Indo-Pacific spiny lobster species, the *Panulirus homarus* (Linnaeus, 1758) species complex has a strong genetic structure along with a very extensive distribution, despite its long larval period (~ 6 months). The species has three or four morphological subspecies (differentiated by color pattern and squamae, or sculpturation pattern in grooves of abdominal tergits) [21, 22] (Supplementary file 1- Fig. S1). Our previous research using mtDNA and ITS-1 sequences, and microsatellite variation, confirmed the genetic difference of the *P. homarus rubellus* subspecies from South African (SA) waters, with the possibility of limited secondary contact with the adjacent *P. h. homarus* subspecies in the Indian Ocean [10]. However, the data refuted the genetic distinctiveness of the morphologically-defined *P. h. megasculpta* samples from the coasts of Iran and Oman in Northwest Indian Ocean (NWIO). Instead, the genetic data indicated that *P. homarus* from each of the East African (EA), NWIO, and core region of the East Indian Ocean/Indo-Australian Archipelago all belonged to the *P. h. homarus* subspecies, with only a small degree of genetic divergence between each region. The small and isolated eastern peripheral population in the Marquesas Islands (MI) in the central Pacific Ocean had previously been identified as another potential subspecies (*P. h. “*Brown*”*), but was identified genetically as merely a divergent remnant population [9, 10]. However, it remains unclear what are the detailed historical genetic relationships among all these regional subspecies and populations (i.e., *P. h. rubellus* from SA, and *P. h. homarus* from its “Central core” region of the East Indian Ocean/Indo-Australian archipelago, and its peripheral populations in EA, NWIO, and MI), or what are the drivers of these potentially complex patterns of divergence.

Here we used genomic SNP and mtDNA sequencing data to test a variety of potential historical models of population divergence, admixture and demographic size change, to determine the most likely evolutionary histories of the *P. homarus* complex throughout its wide distribution. Additionally, we undertook an initial tests of predictions of the “core-periphery hypothesis (CPH) from which it could be anticipated the genetic variation and demographic size of a species’ decreases from the center to the edge of its geographic distribution, along with the degree of connectivity [23] as observed in some other Indo-Pacific marine species [e.g.,7]. This study provides a better understanding of the crucial genetic processes leading to lineage divergence and the maintenance of reproductive isolation in marine organisms before full reproductive incompatibility has been achieved.

## Material and methods

### Sample collection and DArTseq genotyping

**Fig. 1:**
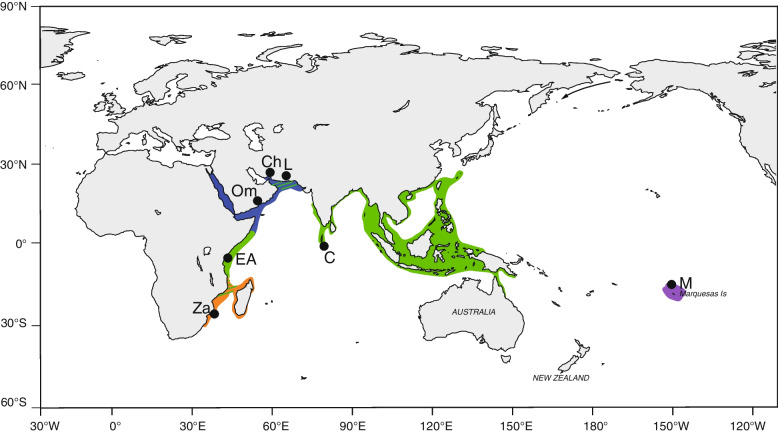
Species distribution and sampling locations for *P. homarus* spiny lobsters in the Indo-Pacific Ocean. Circles are sampling locations in this study and color represents the morphotypes (orange: South Africa *P. h. rubellus* (SA); blue: Northwest Indian Ocean (previously *P. h. megasculpta*) (NWIO), green: Central *P. h. homarus* (C); purple: Marquesas Islands (MI) (previously *P. h.* Brown). Color dashed lines show the hybrid zones

Muscle tissue was taken from the pleopods of lobster specimens sampled from all five major regions across the Indo-Pacific Ocean known to be genetically differentiated from previous studies [9] (Fig. 1, Table 1). Sampling included subtypes from South Africa (SA) in the Southwest Indian Ocean (morphotype described as *P. h. rubellus*), Tanzania in eastern Africa (EA) (morphotype described as *P. h. homarus*), Oman and Iran in the Northwest Indian Ocean (NWIO) (morphotype previously described as *P. h. megasculpta*), from the species’ genetically homogeneous “Central core” (C) distribution throughout the East Indian Ocean/Indo-Australian Archipelago (with representative samples from South India) (morphotype described as *P. h. homarus*), and from the Marquesas Islands (MI) isolated population in the mid-Pacific Ocean (morphotype described as *P. h. “*Brown*”*). The samples were either purchased from fishers or fisheries markets after confirmation of geographic location of capture or obtained by courtesy of research organizations as described in Lavery et al. [10] and preserved in 95% ethanol. SNP discovery used a genotype by-sequencing approach (Diversity Array Technology DArTseq; DArT Pty Ltd. Canberra, Australia) from approximately 40 mg of ethanol preserved muscle tissue from each specimen, as described in Sansaloni et al. [24]. Genomic DNA was digested using a combination of PtsI and HpaII enzymes, and then size selected. Pooled multiplexed libraries (94 individuals and 2 negative controls in each) were sequenced on an Illumina HiSeq2500 platform for 77 cycles.

**Table 1:** Sample sizes sequenced for SNP discovery using DArTseq for each region, along with genetic diversity indices

Location (Abbreviation)	N	MLH	AR	sMLH	F_IS_	H_e_ (±SD)	H_o_ (±SD)
South Africa (SA)	56^a^	0.289	1.25 + 016	1.000 (0.1608)	0.369	0.210 (0.1757)	0.135 (0.1376)
East Africa (EA)	14	0.225	1.21 ± 0.18	0.986 (0.1608)	0.262	0.155 (0.1763)	0.1205 (0.160)
Northwest Indian Ocean (NWIO)	77	0.312	1.27 ± 0.14	1.008 (0.1608)	0.345	0.239 (0.1698)	0.158 (0.1344)
Central (C)	33	0.247	1.22 ± 0.18	0.998 (0.1609)	0.271	0.161 (0.1722)	0.120 (0.1477)
Marquesas Islands (MI)	11	0.050	1.10 ± 0.18	0.998 (0.1608)	0.137	0.059 (0.1490)	0.059 (0.1490)

*N* number of samples, *MLH* multilocus heterozygosity, *AR* allelic richness, *sMLH* standardized multilocus heterozygosity, *F*_*IS*_ inbreeding coefficient, *He* expected heterozygosity, *Ho* observed heterozygosity. Further indices are provided in Supplementary Table S4.^a^ mtCR dataset sample size = 57

### Quality control, genetic structure diversity and analysis

After sequence data analysis, QC and SNP calling by the Diversity Array Technology proprietary pipeline (DArT PL) (see Lind et al. [25]) genotypic data were provided in CSV format. The monomorphic loci and SNPs with call rates of non-missing values below 90% for loci (80% for individuals), minimum depth rate of five and technical repeatability (consistency of marker in technical replicate) less than 95% and were filtered out. Then the dataset was filtered to obtain only one SNP per sequenced tag in dartR package [26]. The hamming distance threshold of 0.25 was applied to filter out paralogous loci also in dartR. Deviation from HWE (*P* < 0.00001) and linkage disequilibrium (r^2^ > 0.80) of loci were pruned in Plink 1.9 [27]. Finally, loci and individuals with more than 10% missing data and minor allele frequency below 2% [12] were also removed using Plink 1.9 prior to further population genetic or demography analysis.

Population genetic diversity indices including, expected (H_E_) and observed (H_O_) heterozygosity and inbreeding coefficient (F_IS_) were estimated by Genetix [28]. Standardized multi-locus heterozygosity (sMLH) was calculated for each population using inbreedR package [29]. Allelic richness (AR) was calculated in diveRsity package in R [30]. Several different methods were used to detect outlier loci, under potential divergent or balancing selection, including BayeScan v2.1 [31], Outflank [32], and FDIST coalescent simulation implemented in Arlequin 3.5 [33], using hierarchical island model and 100,000 simulations. The false discovery rate (FDR) correction of 0.05 was applied to account for multiple testing.

### Phylogeography, admixture, and demographic history analysis

Analysis of molecular variance (AMOVA) and pairwise F_ST_ was performed in R package StAMMP [34] for the whole SNP dataset and for loci putatively under selection. Discriminant analysis of principle components (DAPC) was performed in adegenet package [35] to visualize the genetic structure of populations in multivariate space after finding the optimal number of clusters. A Principal Coordinate analysis (PCoA) was also performed using dartR gl.pcoa function for visually revealing genetic structure. Population admixture and ancestry analysis was carried out in ADMIXTURE program [36] and LEA package [37] with sNMF function. Initially, the most likely number of K was estimated by using a broad range of possible clusters (K = 1–12), and then optimal K with higher confidence (K = 2–5) were run with 10 replicates to find the best K. Regarding the previously reported morphological difference and also those observed in our samples (Supplementary file 1- Fig. S1) and based on our admixture analysis results, we tested the possibility of hybridization events further. The hybrid occurrence and probability among lineages of *P. homarus*, was checked in NEWHYBRIDS [38] using Jeffreys-like prior based on the 300 highest F_ST_ loci. The phylogeographic relationship of individuals from the SNP dataset was represented in a minimum spanning network constructed from Bruvo’s distance in Poppr package [39].

Historical demographic patterns were evaluated using the ∂a∂i package in Python3.8 based on methods described in Gutenkunst et al. [40] using the AFS. An allele SNP matrix was imported into ∂a∂i and the folded frequency spectrum was created. Then various demographic scenarios, including combinations of population split, migration (secondary contact), and population size change were tested on the specific population pairs of interest after finding the best projection for each pair. The EA population was found to be genetically similar to that of the Central (C) region in genetic analysis and were excluded for these analyses. The final comparisons used were *P. h. rubellus* in SA versus the adjacent Indian Ocean/Central *P. h. homarus*; *P. h. megasculpta* in NWIO versus the adjacent Central *P. h. homarus*; and the Central *P. h. homarus* MI. The demographic scenarios compared included a standard neutral model (population split with constant population size over time), population size change, secondary contact, and division into different time periods (see Supplementary file 2 for details). Asymmetric migration rates were considered wherever migration (2N_ref_ migrants per generation) was included in the models. The best scenario was selected using log-likelihood and Akaike information criterion (AIC) and was confirmed by visually comparing the data and model allele frequency spectrum plots. Then the best model’s parameters were optimized using 100 simulations. Further details of model selection and analysis were as described in Silva et al. [16] and Portik et al. [41]. The conversion of the obtained demographic parameters to biological units (N_ref_, mutation rate and generation time) was carried out as per Silva et al. [16]. For comparison, equivalent demographic analyses were also performed using approximate Bayesian computation in DIYABC [42] with the same demographic scenarios simulated for one million summary statistics per scenario. The goodness-of-fit for models with the highest posterior probabilities was estimated using the model checking option in DIYABC.

The maximum likelihood tree haplotypic median-joining network and Bayesian clustering (K = 3 was the best fitting number based on likelihood values) using BAPS [43] of the mitochondrial control region (mtCR) on the same samples for which DArTseq was performed as described in Farhadi et al. [9]. A putative hybrid sample with *P. homarus* mtCR lineage from SA (PhSa78) database failed in SNP genotyping and therefore was only included in mtCR analysis.

## Results

After filtering steps, a total of 2020 SNP loci for 191 individuals were retained for analysis (detail on the number of loci retained in each filtering step and each criterion is presented in Supplementary file 1- Table S1).

Measures of heterozygosity and inbreeding across all surveyed populations (Table 1) indicate that, compared to the large Central Core population (C), the NWIO and SA populations have somewhat elevated heterozygosity (H_o_, H_e_, sMLH & AR) as well as inbreeding (F_IS_), while the MI population has considerably lower values for all these measures. Moreover, the highest values of diversity indices were observed for NWIO and then SA populations. Arlequin and BayeScan identified 181 and 121 outlier loci respectively and those SNPs shared between the results of these two methods (81) were considered as outliers for further analyses. No outlier loci were detected by outflank.

### Genetic structure

The AMOVA test of population structure across the entire SNP data set revealed significant genetic structure (F_ST_ = 0.148, *P* < 0.0001, and outlier F_ST_ = 0.637 versus mtDNA Ф_ST_ = 0.68, P < 0. 001) among the five regional groupings. Pairwise genetic differentiation tests showed the highest difference between MI and all other regions, and the smallest differences between the Central and EA regions, for both data sets (Table 2). The pairwise F_ST_ values between SA *P*. *h*. *rubellus* (including putative hybrid specimens) and all other locations except MI were lower than the divergences observed for the MI, in contrast to the pattern observed in mtCR DNA sequence data (Table 1 and Supplementary file 1- Table S2). Overall, as expected, outlier loci showed greater F_ST_ values than the entire data set, but also showed comparatively greater divergence of the MI.

**Table 2:** Pairwise SNP F_ST_ values between regional populations of *P. homarus*

	SA	EA	NWIO	C	MI
SA	0	**0.2329**	**0.1206**	**0.2124**	**0.5679**
EA	**0.1227**	0	**0.1119**	**0.0325**	**0.3514**
NWIO	**0.072**	**0.0501**	0	**0.0637**	**0.4965**
C	**0.1269**	**0.0171**	**0.0475**	0	**0.4092**
MI	**0.2539**	**0.2302**	**0.1892**	**0.1227**	0

Above diagonal are values from outlier SNPs, below diagonal from the entire SNP dataset. All F_ST_ values were significant after Bonferroni correction (bold)

**Fig. 2:**
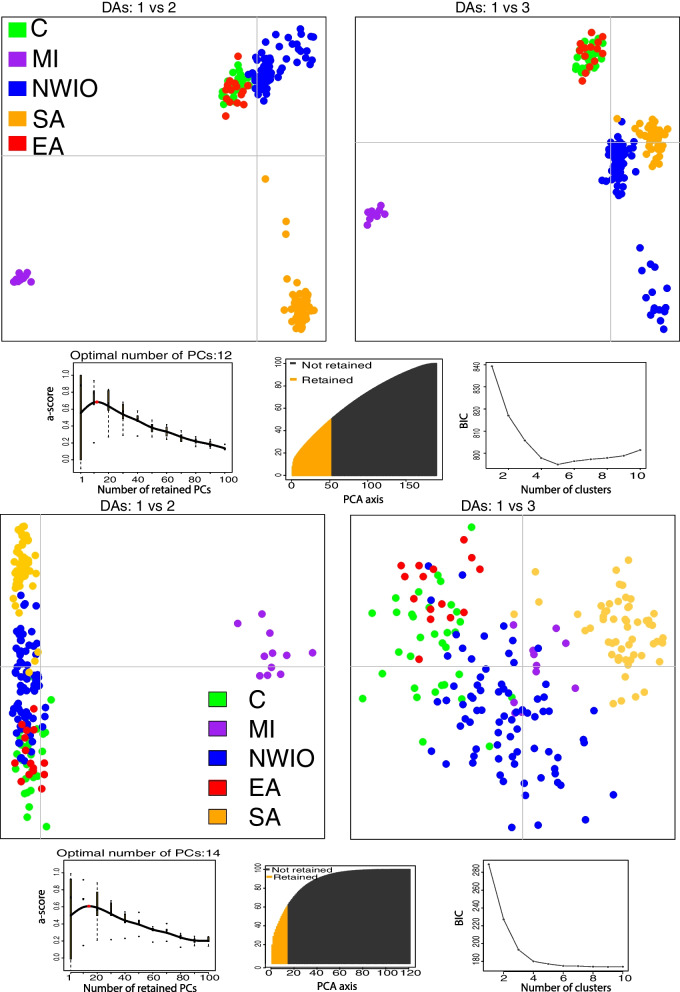
DAPC scatter plot of *P. homarus* main lineages using SNP dataset in this study, a) all SNP dataset b) outlier SNP dataset

All these relationships are also clearly apparent in the DAPC analyses (Fig. 2). Across all loci (Fig. 2a), both peripheral MI and SA populations were clearly differentiated from the others with discriminant analysis (DA) 1 and 2, while DA 2 and 3 clearly differentiated NWIO from EA & C populations. The DAPC analysis of outlier loci revealed some slightly different patterns (Fig. 2b). Comparatively, the MI population was more divergent while the other populations were less differentiated. Furthermore, some *P*. *h*. *rubellus* individuals from SA were more similar to *P*. *h*. *homarus* individuals from the Indian Ocean, and the NWIO individuals appeared as a diverse group of two clusters (Fig. 2b; also PCoA analysis, Supplementary file 1- Fig. S2).

**Fig. 3:**
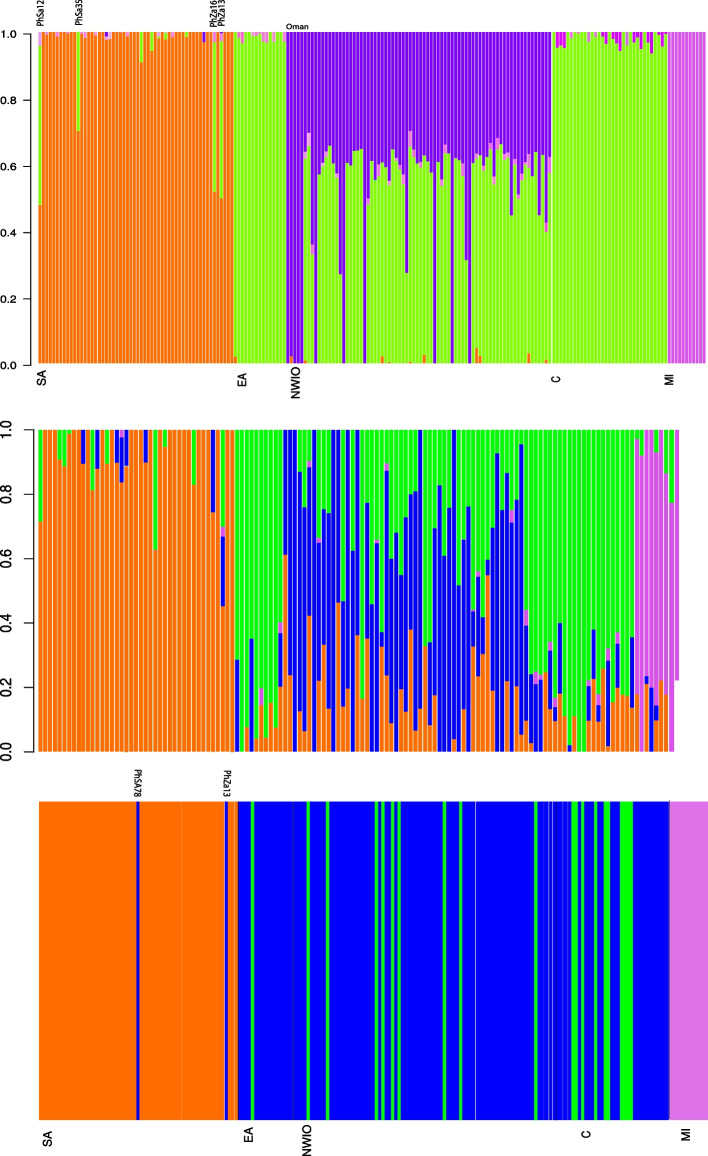
Bar-plot of admixture (top, at optimal K = 4), admixture bar-plot of outlier loci (middle, k = 4), mtCR BAPS plot (bottom, optimal K = 3) on *P. homarus* populations. Each vertical bar represents an individual

**Fig. 4:**
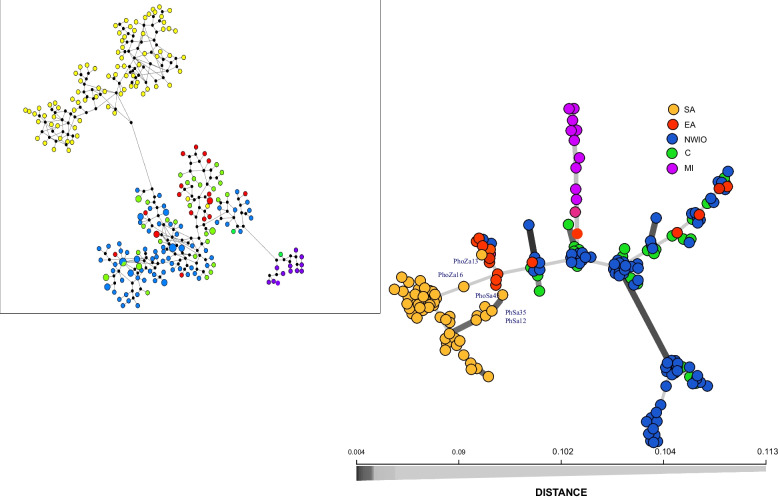
Minimum spanning network representing the phylogeographic relationship between *P. homarus* lineages, left: using genome wide SNPs, right: using mtCR

Assignment analyses of SNP and mtDNA data (Fig. 3) revealed strong population clustering, both with similar results seen in the most peripheral populations (SA and MI), but differences among the Indian Ocean populations. Both admixture (Fig. 3a) and LEA analyses (Supplementary file 1- Fig. S2) of SNP data revealed an optimal clustering of k = 4. Both the MI and SA populations were generally well-differentiated from the other populations, but a number of SA individuals appear to have mixed ancestry with the adjacent main *P*. *h*. *homarus* cluster. Within the other Indian Ocean populations, the individuals from NWIO appear to have a largely mixed ancestry between a genetic lineage found in the NWIO and Central populations, and a second lineage which constitutes the entire ancestry of a small number of individuals (total of 11 samples) sampled from both Oman and Iran (Fig. 3 and Supplementary file 1- Fig. S3). In contrast, the mtDNA (Fig. 3b) and previous microsatellite analysis [9], revealed only that the EA, NWIO and C populations were a single cluster with some mixed origin. These relationships are somewhat clarified with the help of minimum spanning networks depicting the relationships among genomic genotypes and mtDNA haplotypes (Fig. 4). In the mtDNA network, it is only the SA and MI populations that demostrate clear phylogeographic divergences, while all other populations appear mixed. In contrast, in the SNP network, the SA and MI populations are still largely distinct, but the remaining individuals appear to fall into two phylogeographic lineages: one found only in NWIO, and a second diverse lineage found in EA, NWIO and C populations. These relationships are also shown in more detail in ML phylogenetic trees (Supplementary file 1- Fig. S4).

### Secondary contacts among lineages

Individual-based analyses of potential hybridization (undertaken using NewHybrid) revealed a number of individuals with likely hybrid origins (Table 3). Four individuals from SA, all morphologically identified as *P. h. rubellus*, were shown to have high confidence as being hybrids between the *P. h. rubellus* and *P. h. homarus* genomic lineages, one individual with hybrid morphotype (Fig. S1) from SA was also genetically confirmed as a hybrid using SNPs. These individuals were variously assigned to most likely be derived from F1 or F2 genetic backgrounds and had either *P. h. rubellus* or *P. h. homarus* mtDNA (The detailed comparison of the alternate SNP and mtDNA lineages of these individuals can be seen in Fig. S2). Furthermore, the majority of individuals from NWIO were shown to have a hybrid origin between the genomic lineage found in NWIO and Central populations (supplementary file 1-Fig. S3), and the genomic lineage found in several NWIO individuals from Oman (e.g., PhOm02, see Table 3). Each of these hybrid individuals were identified to have a more complex hybrid background, including proportions of F1, F2 and substantial backcrossing.

**Table 3:** Potential hybrid individuals and their estimated genetic background. Only a few representative hybrids from NWIO are listed, as most NWIO samples are admixed

Individual^a^	NewHybrid result^b^	Admixture clustering^c^	mtDNA^d^
C is P0 and SA is P1			
PhZa13	F1	*P. h. homarus*50%-*P. h. rubellus*50%	*P. h. homarus*
PhZa16	F2	*P. h. homarus*50%-*P. h. rubellus*50%	*P. h. rubellus*
PhSa12	F1P1	*P. h. homarus*30%-*P.h.rubellus*70%-	*P. h. rubellus*
PhSa35	F2	*P. h. rubellus*70%- *P. h. homarus* 30%	*P. h. rubellus*
PhSa77^e^	–	–	*P. homarus*
C is P0 and NWIO is P1			
PhCc04	F1P1	Central%30-NWIO%70	*P. homarus*
PhCc12	45%F2–55%F1P1	Central-%50-NWIO%50	*P. homarus*
PhCd10	100%P1	NWIO100%	*P. homarus*
PhCd19	41%P0–29%F2–30%F2P0	Central-%50-NWIO%50	*P. homarus*
PhCr19	32%P1–26%F2–42%F1P1	Central%50-NWIO%50	*P. homarus*
PhL10	F2	Central%50-NWIO%50	*P. homarus*
PhOm02^f^	100%P1	NWIO100%	*P. homarus*

^a^ Sampling codes: Za, Sa – South Africa; Cc, Cr, L, Cd, Om (Oman) – NWIO locations

^b^ Posterior probabilities of belonging to different category genetic backgrounds: P1 – pure pop 1; P2 – pure pop. 2; F1 – F1 hybrid; F2 – F2 hybrid; FxPx: backcross of Fx hybrid to population x

^c^ Assignment probabilities to different genetic clusters, determined by admixture LEA analysis

^d^ mtDNA clade

^e^ DArTseq genotyping for this sample was not successful

^f^ PhOm02 and PhCd10 are representatives pure NWIO-specific lineage

### Analysis of demographic inference

**Fig. 5:**
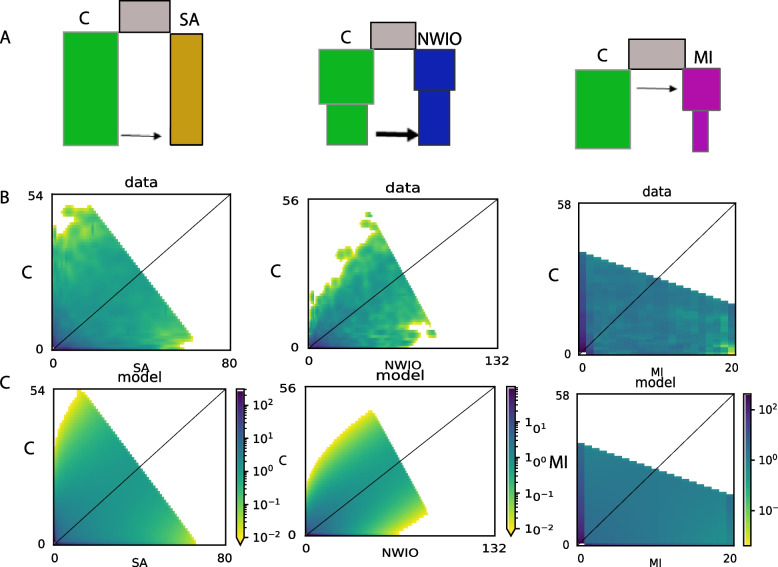
∂a∂i best-fit models for S. Africa-Central (SA-C), NWIO-Central (NWIO-C), and Central-Marquesas Islands (C-MI) population pairs. **A** Demographic model diagrams indicating relative time since population split, relative timing and direction of asymmetric gene flow (> 10-fold difference in m), and relative population sizes. **B** Joint allele frequency spectra (AFS) obtained from data. **C** joint AFS obtained from the best fit model. Further detail in Supplementary file 1- Fig. S6 and Table S3

The demographic simulations of the histories of the three divergent peripheral populations (SA, NWIO and MI), undertaken in ∂a∂i, showed that a range of different demographic events best fitted the genomic SNP data (Fig. 5, Table 4). These events included ancient asymmetric migration, recent secondary contact, and recent population size changes. Overall, the best-fitting demographic models for the population pairs were as follows (Fig. 5, supplementary file 1- Fig. S5); SA-Central: after a split from the ancestral population, a long period of isolation, followed by recent secondary contact with asymmetric migration into SA (divergence time ~ 100 x secondary contact time). NWIO-Central: a similar model, but with recent asymmetric secondary contact accompanied by a population size reduction. Central-MI: after split, early asymmetric migration to MI, followed by isolation and population size reduction. Based on the ∂a∂i analyses, the SA *P. h. rubellus* was the earliest population to diverge from the Central *P. h. homarus* lineage. The relative divergence times estimated from the demographic models were (Table 4): divergence of *P. h. rubellus* and *P. h. homarus* 14.9*2N_ref_ (ancestral theta = 481) generations ago; NWIO *P. h megasculpta* and *P. h. homarus*- 30*2N_ref_ (ancestral theta = 293) generations ago, Marquesas – *P. h. homarus*- 0.2*2N_ref_ generations ago. Each of the asymmetric migration rates were greater than ten times that estimated for the reverse direction. Each of the peripheral populations were estimated to have population sizes less than one half that of the Central population after initial demographic split, and both the NWIO and MI populations were estimated to have had recent population size reductions. Demographic analyses using DIYABC resulted in remarkably similar best-fitting demographic histories (Supplementary file 1- Fig. S5). The *P. h. rubellus* population was estimated to be the earliest diverged, with a long period of isolation before very recent secondary contact. The DIYABC analysis estimated that this recent introgression was more limited in extent and occurred in both directions (Supplementary file 1 - Fig. S5). Again, the demographic history estimated for the NWIO population was similar, except that two pulses of immigration were estimated, one ancient and one very recent. Finally, the estimated history of the Marquesas population was also very similar to that from ∂a∂i, with no recent immigration. The DIYABC analyses concurred that the peripheral populations all had lower population sizes than that of the Central population.

**Table 4:** Parameter values for best fitting demographic models

Pop pair	C_SA	C_NWIO	C-MI	NWIO_SA
Model Name^a^	Snomig_secont	Secont_asymigsize	S_asymig_size	Snomig-size
Parameters^b^:				
Theta	481	293	479	630
nu1	0.26	15.50	1.98	4.42
nu2	0.10	4.69	0.82	0.99
nu1b	–	0.87	2.02	1.61
nu2b	–	0.87	0.33	0.62
m12	2.15	1.07	1.96	–
m21	22.39	11.90	19.98	–
T1	14.91	30.13	0.23	1.78
T2	0.148	0.17	0.18	0.25

^a^: S- split

Asymig - asymmetric migration

nomig - no migration

secont - secondary contact

size - pop. Size change

^b^: Theta: for ancestral population before split (θ = 4N_ref_μ)

nu1: Size of population 1 after split. nu2: Size of population 2 after split

nu1b: Size of population 1 after change. nu2b: Size of population 2 after change

m12: Migration from pop 2 to pop 1 (2*Na*m12). m21: Migration from pop 1 to pop 2

T1: Time of split (2Ne generations in past). T2: Time of secondary contact/pop. Size change (2Ne generations in past)

## Discussion

The application of SNPs for the analysis of genomic diversity in the *P. homarus* complex has facilitated new insights into the evolutionary processes occurring in this species. Overall, the analyses reveal that this species complex exhibits a rich and dynamic pattern of historic and present divergences and introgression, shedding light on the diverse evolutionary processes that contribute to high marine species diversity in the Indo-Pacific. The study confirmed an instance of mitonuclear discordance using both genome-wide SNPs and mitochondrial DNA sequences, highlighing the advantage of using genome-wide SNPs coupled with mitochondrial DNA to help understanding population admixture.

### Patterns of genetic diversity and structure

Overall, the genetic diversity values observed within *P. homarus* from SNP data were similar to those observed in other spiny lobsters [12, 44]. The overall reduced levels of observed versus expected heterozygosity within populations, although not significant, have led to generally raised F_IS_ values. The patterns across populations fit the overall population structure interpretations (see below), with both higher diversity and homozygosity in populations believed to be of mixed origin (SA & NWIO, where high F_IS_ may be due to a Wahlund effect), and lower diversity and F_IS_ in the isolated, remnant population (MI). Similar raised F_IS_ has been observed in other crustacean studies, including on *P. ornatus* [45], *P. homarus* within NWIO [12] and the widely distributed blue swimmer crab, *Portunus pelagicus* [46].

The genetic diversity across the population groupings of *P. homarus* is at its lowest in the eastern peripheral population (MI), but highest in two of the western peripheral populations (SA & NWIO), a pattern consistent with the CPH which predicts that genetic diversity should be higher in the central population than at the margins. However, the CPH is not always a reliable explanation for the patterns of genetic variation within populations including when there are a lower amounts of sample date from the margins of the population range for comparison, as in this study. Other factors are acting in the western periphery of this species, where secondary contacts appear to have occurred between core and peripheral populations may explain the observed genetic diversity pattern. Another marine species with similarly wide Indo-Pacific distribution, the pearl oyster (*Pinctada margaritifera*) shows similar partial support for CPH hypothesis, but instead at the western margins [47].

AMOVA and pairwise F_ST_ analyses revealed very strong genetic structure in the *P. homarus* complex. The results confirmed the patterns of strong genetic divergence observed in mtDNA data in the previous study [9], and revealed a common pattern of three main lineages (SA *P. h. rubellus*, MI and the rest of *P. homarus*) (Fig. 4b). The results also provided further genetic detail to help unravel the complex relationship between the C and NWIO populations (DAPC, admixture, and genotype network; Figs. 2, 3, 4), overturning some of the recent genetic conclusions about the NWIO population.

The new genomic analyses shed an entirely new light on the NWIO population, once again providing support for the existence of a distinct *P. h. megasculpta* genetic lineage, and explaining the inconsistencies in previous genetic data that questioned the genetic basis of this subspecies from this NWIO region. Previously, a distinct morphotype and subspecies (*P. h. megasculpta*) had been described [21, 22] in this region. However, more recent genetic analyses using mitochondrial and nuclear DNA (16S, COI, control region and ITS-1) loci, and nuclear microsatellite data did not reveal a distinct lineage in samples from this region [9, 10]. Instead, there appeared to be a degree of population-level genetic differentiation, based on differing mtDNA haplotype and nuclear allele frequencies, although the observed degree of differentiation appeared to vary depending on the sampling locations used [9, 48, 49].

### Evolutionary demographic history

The historical demographic analyses provide a much stronger evolutionary context for interpreting the genetic introgression between the *P. h. homarus* and NWIO lineages. Both the ∂a∂i and ABC analyses indicate that the NWIO population has experienced a long period of genetic isolation from the remainder of *P. h. homarus*, with only very recent secondary contact driving the clear pattern of introgression observed.

Dating these evolutionary events accurately is difficult without a much stronger knowledge of rates of SNP mutation in this species. However, the mtDNA data indicates the (now apparent) “NWIO lineage” was estimated to have diverged between 0.6 mya [9] and 6 mya [48], depending on calibration. Using an estimated SNP mutation rate (8*10^− 8^) and generation time used by Silva et al. [16], our demographic analysis estimated a divergence date of 1000 kya for the NWIO lineage, with a date of secondary contact around 16 kya.

The SNP analyses confirmed the divergence between the South African *P. h. rubellus* population and the adjacent *P. h. homarus*. From all the DAPC, admixture, network, demographic and hybrid analyses (Figs. 2, 3, 4, 5, Table 4) *P. h. rubellus* has consistently been identified as having diverged a long time in the past, with very recent secondary contact leading to limited hybridisation with *P. h. homarus* from the adjacent EA coast. The SNP data has added to our understanding of the extent, and degree of hybridisation.

The SNP genotype network and demographic analyses indicate that the *P. h. rubellus* lineage has been isolated for a very considerable period of time. The previous mtDNA analyses suggest that this is somewhere between 3 mya [9] and 25 mya [48], depending on calibration, while our SNP estimate is around 2 mya. The demographic analysis suggests that secondary contact has been very recent (around 20 kya).

The introgression between the *P. h. homarus* and NWIO lineages appears to have been more prominent throughout the Iranian distribution in NWIO compared to the Oman distribution (separated by the Gulf of Oman), which appears to have experienced comparatively little hybridization. The apparent relative isolation of this portion of the Indo-Pacific range of this species has been previously identified for this species [12, 48, 49] as well as for a number of other marine species (e.g., Torquato et al. [50]). A series of strong seasonal eddies and upwellings along the coasts of Yemen and Oman are known to act as a barrier to genetic connectivity, and also promote the retention of locally-produced pelagic larvae [49, 50]. It appears that changing patterns of currents over long periods of time have led alternately to dispersal of *P. homarus* to this NWIO region, followed by a long period of isolation, favored by oceanographic conditions in the Miocene and Pliocene, and then recent post-glacial conditions that may have favored reconnection and secondary contact.

The generally isolated nature of the NWIO over the long term was likely due to monsoonal upwellings (over the last ~ 13 mya) along the Somali and Oman coastlines, and is exemplified by the existence of a significant and persistent Oxygen Minimum Zone (OMZ) in the Arabian Sea over this long period [51]. Although the OMZ is generally a mid-water phenomenon, it can be pronounced in the NWIO at even 25 m depth during periods of the year [52]. There is evidence that this OMZ was considerably strengthened during the period from late Miocene (~ 13 mya) until the Pleistocene [51]. During this period, the Arabian Sea appears to have been somewhat isolated by Indian Ocean currents, permitting genetic isolation in *P. homarus*. In addition, the marine environmental conditions throughout the OMZ in this region were considerably different from much of the surrounding Indian Ocean waters, likely providing strong selection pressures favouring the adaptive divergence of marine species in these waters. Both these factors are likely to have favored the divergence of a distinct lineage (or even subspecies) in this NWIO region. Evidence supporting the marine distinctiveness of the NWIO region includes its recognition as a distinct biogeographic province [53] and its recent identification as a distinct phylogeographic region in a reef fish using SNPs [54].

A substantial reduction of the monsoonal upwellings along the western Indian Ocean coastline (the Somali & Oman upwellings) occurred during the Pleistocene glacial maxima, prompting a decrease in the intensity of the OMZ over these periods [51], and allowing greater mixing of the waters of the Arabian Sea with the broader Indian Ocean waters [55]. This change in the OMZ along with the strengthening of South Asian Monsoon [56], would have changed current patterns in the region during the Pleistocene glacial maxima, likely increasing dispersal of *P. homarus* phyllosoma in the northern Indian Ocean and facilitating recent admixture between the Central (*P. h. homarus*) lineage and the NWIO (*P. h. megasculpta*) lineage in the NWIO region. There is recent evidence from fish species/subspecies with restricted distributions in the Arabian Sea, that these isolated lineages have occasionally mixed with adjacent endemic lineages to form hybrid populations in the NWIO [50, 57, 58].

### Hybrid origin of the subspecies

In the mtDNA data, NWIO individuals from Oman and Iran had haplotypes scattered throughout the mtDNA phylogenetic tree/network, although a number of individuals sampled from Oman clustered together in their own clades [8–10]. In contrast, where NWIO individuals were sampled only from the Yemeni/Omani coast, their mtDNA haplotypes fell mostly, but not entirely, into a distinct lineage [48]. Similarly, NWIO individuals were much more clearly assigned to a distinct microsatellite genetic cluster when they were derived from only the Yemeni/Omani coast [49], than when they were derived from both Oman and Iran [9]. Although the previous genetic data is in fact consistent with the new interpretation, without the clear evidence of a distinct nuclear lineage and introgression from the present SNP data, the distinctiveness of the NWIO population and the potential legitimacy of the *P. h. megasculpta* subspecies could not be supported.

All SNP analyses (PCoA, DAPC, network, and assignment, supplementary file 1- Figs. S3 & S4, Figs. 2, 3 and 4) clearly indicate the existence of strong introgression between two distinct genetic lineages: one from the neighboring *P. h. homarus* populations, but a new, distinct lineage found only in NWIO, the “NWIO lineage”. The admixture of genetic material from these two lineages is mostly clearly revealed in the admixture plots (Fig. 3, Supplementary file 1- Fig. S3), which clearly show that most NWIO individuals have a mixture of both these lineages. The admixture plot suggests many of these sampled individuals may be F1 hybrids between the two lineages, but more detailed hybrid analyses (undertaken in NewHybrid, Table 3) reveal that there are a wide range of genetic backgrounds in individuals from this region. These range from having a “pure” NWIO lineage, to various combinations of F1, F2 and backcrosses with the *P. h. homarus* lineage, and noticeably no “pure” *P. h. homarus* lineage individuals. The “pure” NWIO lineage individuals appear to be concentrated mostly in the samples from Oman in NWIO, suggesting that admixture may be affected by localized differences in abundance of individuals representing the two lineages, or localized differences in genetic connectivity.

Unlike that observed in NWIO, only a very small proportion of *P. h. rubellus* individuals were found to be hybrids (Fig. 3). The apparently relatively recent and transitory nature of this hybridization event, along with the very abrupt geographic demarcation between the subspecies, indicates that *P. h. rubellus* has achieved some level of reproductive isolation. Most evidence points to introgression occurring predominantly into *P. h. rubellus*, but the genomic evidence appears to confirm that at least some F1 hybrids are fertile, and continue to pass on some *P. h. homarus* nuclear lineage into *P. h. rubellus*. Given our current understanding that this subspecies is likely adapted to the cooler water temperatures within its range, it will be beneficial to further explore this subspecies for molecular evidence of adaptive traits, the spatial pattern of the presence of hybrids and possible presence of long distance adult migrations.

Previous microsatellite and mtDNA analyses of *P. h. homarus* individuals from just north of the contact zone [48, 49] reveal little evidence of hybridisation. The ∂a∂i demographic analysis suggests that most genetic introgression is into *P. h. rubellus*, although the DIYABC analysis could not rule out that there could be a small degree in both directions.

It appears that most hybridisation events in *P. h. rubellus* individuals were also relatively recent, with detected hybrids most likely being F1 or F2 as well as back cross (Table 3). One South African individual (PhZa13, Figs. 3, S1) has mtDNA from the alternate *P. h. homarus* lineage and was identified as being an F1 hybrid (from NewHybrid analysis), having a 50% SNP admixture of *P. homarus* and *P*. *h. rubellus*, confirming that is it very likely to be an F1 hybrid of a female *P. h. homarus* with male *P. h. rubellus*. Other SA individuals (PhZa16, PhSa12, PhSa35, Fig. 3 and Table 3) have mtDNA from the *P. h. rubellus* lineage and were identified as having variable hybrid SNP admixtures (F1 or F2, 50–70% *P. h. rubellus* SNP contribution), indicating that they are progeny of male *P. homarus* and female *P. rubellus*. The demographic modeling provided additional evidence of the recent admixture of the *P. homarus* lineage into *P*. *h*. *rubellus* (Fig. 5). A recent study with microsatellite markers in the Western Indian Ocean also has reported a degree of admixture between *P. h. homarus* and *P. h. rubellus* in the Mozambique Channel region [49]. The existence of a low level of admixture between these two subspecies appears to be due to some type of strong, persistent, but partly permeable barrier to dispersal along the southeast African coast acting in this region, which was suggested to be the Delagoa Bight Eddy [49]. This barrier may have arisen during the late Miocene, when the final uplift of Madagascar, and volcanic activity along the Dave Ridge off the East African coast, tightened the current flow along the coast and formed the strong Mozambique eddies [59]. This barrier to gene flow appears to have recently become semi-permeable since the LGM, through weakening during glacial phases. The periodic change in upwelling cells and the freshwater plumes of large rivers may have also played a role [60] in recent increased permeability of this barrier between *P. h rubellus* and *P. h. homarus*.

### Speciation patterns in marine invertebrates

Previous genetic examinations of patterns of marine species divergence in the Indo-Pacific have concentrated on either long-diverged species (e.g., *Jasus* spiny lobster: [16]) or very recently diverged populations within a species (e.g., oyster [47]). Although informative, neither type of study captures the genetic processes occurring during speciation itself, nor how new species barriers persist during range expansion of adjacent species, when introgression becomes more likely. The *P. homarus* complex, instead, offers us a valuable insight into the genetic interactions that occur in a species actively undergoing speciation, with perhaps three incipient species forming or being subsumed. It shows that marine genetic patterns are likely to often be more complex than the simple theoretical predictions, such as those of the CPH. This species complex highlights some of genetic processes that are likely to occur in marine species in this region, and addresses the ongoing conundrum of high rates of marine speciation despite very wide dispersal [61].

Perhaps unsurprisingly for a species with such a long pelagic dispersal, most of the subspecies divergence in *P. homarus* is occurring at the peripheries of the distribution. The processes demonstrated here may be likely to occur in many other Indo-Pacific marine species with wide larval dispersal, although at different geographic scales, dependent on their maximum dispersal distances.

At one extreme, in the eastern periphery of *P. homarus* complex, there is an apparently relatively simple example of rare dispersal, with a degree of genetic bottleneck, followed by allopatric divergence. The potential physical reasons for MI population’s colonization and subsequent isolation have been discussed previously, but in brief, rely on historical changes in current patterns in the central Pacific [9]. Although there is no explicit evidence of genetic adaptation in this population, it is clear that there has not been any gene flow for a considerable period of time, the outlier analysis shows that loci potentially under selection have diverged most in this population (Fig. 2b), and its morphology has diverged to the extent that is has previously been nominated as a potential subspecies (*P. homarus “*Brown*”*; George, 2006). Although it appears to contain mainly a subset of the genetic diversity of the Central population, without further gene flow it is likely to form a new species in due time.

At the other extreme, at the far western periphery, is a long-diverged population, that has apparently had time to evolve adaptations to cooler waters, and a degree of reproductive isolation. As discussed previously [9], this colonization and isolation is likely due to temporal changes in the strength and eddies of the predominant westerly-flowing Agulhas Current. Recent weakening of these isolating forces appears to have led to a north-easterly expansion of the species range, creating a contact zone with its adjacent forebear population. The subsequent introgression may have interrupted this species’ journey towards full reproductive isolation, but the continuing limited extent of this introgression appears likely to support a continued path towards eventual speciation. A more detailed examination of evidence for molecular adaptation may prove extremely fruitful.

In between these extremes lies the NWIO (*P. h. megasculpta*) population. With the aid of genomic SNP analyses, we now see that this population was likely founded through a rare colonization event more recently than that of *P. h. rubellus*. After a subsequent long period of isolation, sufficient for the emergence of a distinct genetic lineage, it has experienced recent secondary contact at a far earlier stage in its divergence than has *P. h. rubellus*. This has led to extensive introgression, and even though this lineage had diverged sufficiently to establish a distinct morphology, there is clearly little reproductive isolation. It remains to be seen whether this distinct lineage persists, or whether it becomes reabsorbed into the wider *P. h. homarus* population. Perhaps a close parallel can be seen in the largely sympatric and closely-related *P. ornatus*. In this species, there exists a distinct mtDNA lineage that predominates in the western Indian Ocean, where it likely originated, but is now also found at lower frequencies much more widely to the east [11, 45]. We speculate that this could be the future fate of the current *P. h. megasculpta* lineage, if current levels of introgression persist.

There are a number of other examples of widely-distributed marine invertebrates with pelagic larval dispersal that have surprisingly divergent populations in the Western Indian Ocean, which were not predicted from morphology. These include the black-lipped pearl oyster, *Pinctada margaritifera* [47], swimming blue crab, *Portunus segnis* [62] and the pronghorn spiny lobster, *Panulirus penicillatus* [5] all with different degrees of divergence in their western peripheral populations. At least one of the genetic patterns seen within the *P. homarus* complex is likely to have been involved in these divergences, although it now difficult to discern in these species after long periods of divergence.

We suggest that the *P. homarus* complex is an excellent direct illustration of the active isolation and contact during the speciation process in widely distributed marine organisms. Similar patterns of isolation and secondary contract have been reported as occurred among *Jasus* spiny lobsters [16]. For most species examined genetically, these processes are likely to have just begun, or we see only the final genetic end-products, but in *P. homarus* the processes are still in action and observable, which provides a better insight into some of the potential drivers of marine biodiversity in the Indo-Pacific.

## Conclusion

The *P. homarus* complex appears to provide an excellent demonstration of a range of speciation processes that are likely to be common-place in other widely distributed marine invertebrates. Rather than support the simplistic predictions of theories such as the CPH, the genetic patterns observed here strongly suggest that relatively complex historical demographic events have driven the current patterns of marine biodiversity we see in this region. This study also has implications for the appropriate future management and aquaculture of spiny lobsters as a valuable fisheries resource. There is likely to be great value in using further genomic studies of this species complex to identify and understand the role of environmental factors in adaptive speciation. Lastly, it is likely there is similar complexity in the lineage divergence in this species in the South China Sea region and therefore further genomic investigations with larger samples sizes from this region are warranted.

## Supplementary Information

**Additional file 1.** The result of supportive analysis from of mtCR sequences and SNPs in this study.

**Additional file 2.** Script of models used in this study for demographic analysis.

## Data Availability

The genome-wide SNP genotypic data (2020 loci) of *P. homarus* data created and used in this study is available as Supplementary file in STRUCTURE format. The mtCR sequence data are available in NCBI GenBank under accession numbers: KX357386-KX357616, KC625333–KC625469 and KF906454-KF906482. Direct link to sequences NCBI; https://www.ncbi.nlm.nih.gov/popset?DbFrom=nuccore&Cmd=Link&LinkName=nuccore_popset&IdsFromResult=1189429752

## References

[1] Cowman PF, Parravicini V, Kulbicki M, Floeter SR (2017). The biogeography of tropical reef fishes: endemism and provinciality through time. Biol Rev.

[2] Cowman PF, Bellwood DR (2013). Vicariance across major marine biogeographic barriers: temporal concordance and the relative intensity of hard versus soft barriers. Proc R Soc B.

[3] Bernardi G, Bucciarelli G, Costagliola D, Robertson DR, Heiser JB (2004). Evolution of coral reef fish Thalassoma spp. (Labridae). 1. Molecular phylogeny and biogeography. Mar Biol.

[4] Ahti PA, Coleman RR, Dibattista JD, Berumen ML, Rocha LA, Bowen BW (2016). Phylogeography of indo-Pacific reef fishes: sister wrasses *Coris gaimard* and *C*. *cuvieri* in the Red Sea, Indian Ocean and Pacific Ocean. J Biogeogr.

[5] Iacchei M, Gaither MR, Bowen BW, Toonen RJ (2016). Testing dispersal limits in the sea: range-wide phylogeography of the pronghorn spiny lobster *Panulirus penicillatus*. J J Biogeogr.

[6] Phillips BF, Melville-Smith R, Kay MC, Vega-Velázquez A, Phillips BF (2013). *Panulirus* species. *Lobsters: biology, management, Aquaculture & Fisheries: second edition* (second).

[7] Chow S, Jeffs A, Miyake Y, Konishi K, Okazaki M, Suzuki N, Abdullah MF, Imai H, Wakabayasi T, Sakai M (2011). Genetic isolation between the western and eastern pacific populations of pronghorn spiny lobster *Panulirus penicillatus*. PLoS One.

[8] Farhadi A, Farhamand H, Nematollahi MA, Jeffs A, Lavery SD (2013). Mitochondrial DNA population structure of the scalloped lobster *Panulirus homarus* (Linnaeus 1758) from the West Indian Ocean. ICES J Mar Sci.

[9] Farhadi A, Jeffs AG, Farahmand H, Rejiniemon TS, Smith G, Lavery SD (2017). Mechanisms of peripheral phylogeographic divergence in the indo-Pacific: lessons from the spiny lobster *Panulirus homarus*. BMC Evol Biol.

[10] Lavery SD, Farhadi A, Farahmand H, Chan T-Y, Azhdehakoshpour A, Thakur V, Jeffs AG (2014). Evolutionary divergence of geographic subspecies within the scalloped spiny lobster *Panulirus homarus* (Linnaeus 1758). PLoS One.

[11] Yellapu B, Jeffs A, Battaglene S, Lavery SD (2017). Population subdivision in the tropical spiny lobster *Panulirus ornatus* throughout its indo-West Pacific distribution. ICES J Mar Sci.

[12] Al-Breiki RD, Kjeldsen SR, Afzal H, Al Hinai MS, Zenger KR, Jerry DR, Al-Abri MA, Delghandi M (2018). Genome-wide SNP analyses reveal high gene flow and signatures of local adaptation among the scalloped spiny lobster (*Panulirus homarus*) along the Omani coastline. BMC Genomics.

[13] Le Moan A, Gagnaire P-A, Bonhomme F (2016). Parallel genetic divergence among coastal-marine ecotype pairs of European anchovy explained by differential introgression after secondary contact. Mol Ecol.

[14] Ellis CD, Jenkins TL, Svanberg L, Eriksson SP, Stevens JR (2020). Crossing the pond: genetic assignment detects lobster hybridisation. Sci Rep.

[15] Benestan L, Quinn BK, Maaroufi H, Laporte M, Clark FK, Greenwood SJ, Rochette R, Bernatchez L (2016). Seascape genomics provides evidence for thermal adaptation and current-mediated population structure in American lobster (*Homarus americanus*). Mol Ecol.

[16] Silva C, Murphy N, Bell J, Green B, Duhamel G, Cockcroft A, Hernández C, Strugnell J (2021). Global drivers of diversification in a marine species complex. Mol Ecol.

[17] Wang O, Somogyi S, Ablett R (2018). General image, perceptions and consumer segments of luxury seafood in China. Brit Food J.

[18] Spanier E, Lavalli KL, Goldstein JS, Groeneveld JC, Jordaan GL, Jones CM, Phillips BF, Bianchini ML, Kibler RD, Díaz D, Mallol S, Goñi R, Van Der Meeren GI, Agnalt AL, Behringer DC, Keegan WF, Jeffs A (2015). A concise review of lobster utilization by worldwide human populations from prehistory to the modern era. I ICES J Mar Sci.

[19] Boavida-Portugal J, Rosa R, Calado R, Pinto M, Boavida-Portugal I, Araújo MB, Guilhaumon F (2018). Climate change impacts on the distribution of coastal lobsters. Mar Biol.

[20] Fitzgibbon QP, Battaglene SC, Jeffs AG (2014). The Achilles heel for spiny lobsters: the energetic of the non-feeding post-larval stage. Fish Fish.

[21] Berry PF (1974). A revision of the *Panulirus homarus*-group of spiny lobsters (Decapoda, Palinuridae). Crustaceana..

[22] George RW (2005). Tethys Sea fragmentation and speciation of *Panulirus* spiny lobsters. Crustaceana..

[23] Pironon S, Papuga G, Villellas J, Angert AL, García MB, Thompson JD (2017). Geographic variation in genetic and demographic performance: new insights from an old biogeographical paradigm. Biol Rev.

[24] Sansaloni C, Petroli C, Jaccoud D, Carling J, Detering F, Grattapaglia D, Kilian A (2011). Diversity arrays technology (DArT) and next-generation sequencing combined: genome-wide, high throughput, highly informative genotyping for molecular breeding of *Eucalyptus*. BMC Proc.

[25] Lind CE, Kilian A, Benzie JAH (2017). Development of diversity arrays technology markers as a tool for rapid genomic assessment in Nile tilapia, *Oreochromis niloticus*. Anim Genet.

[26] Gruber B, Unmack PJ, Berry OF, Georges A (2018). dartR: an R package to facilitate analysis of SNP data generated from reduced representation genome sequencing. Mol Ecol Resour.

[27] Chang CC, Chow CC, Tellier LC, Vattikuti S, Purcell SM, Lee JJ (2015). Second-generation PLINK: rising to the challenge of larger and richer datasets. GigaScience.

[28] Belkhir K, Borsa P, Chikhi L, Raufaste N, Bonhomme F (2015). GENETIX 4.05, logiciel sous Windows TM pour la génétique des populations (4.05).

[29] Stoffel MA, Esser M, Kardos M, Humble E, Nichols H, David P, Hoffman JI (2016). inbreedR: an R package for the analysis of inbreeding based on genetic markers. Methods Ecol Evol.

[30] Keenan K, Mcginnity P, Cross TF, Crozier WW, Prodöhl PA (2013). diveRsity: an R package for the estimation and exploration of population genetics parameters and their associated errors. Methods Ecol Evol.

[31] Foll M, Gaggiotti O (2008). A genome scan method to identify selected loci appropriate for both dominant and codominant markers: A Bayesian perspective. Genetics.

[32] Whitlock MC, Lotterhos KE (2015). Reliable detection of loci responsible for local adaptation: inference of a null model through trimming the distribution of FST. Am Nat.

[33] Excoffier L, Lischer HEL (2010). Arlequin suite ver 3.5: a new series of programs to perform population genetics analyses under Linux and windows. Mol Ecol Resour.

[34] Pembleton LW, Cogan N, Forster JW (2013). StAMPP: an R package for calculation of genetic differentiation and structure of mixed-ploidy level populations. Mol Ecol Resour.

[35] Jombart T, Ahmed I (2011). Adegenet 1.3-1: new tools for the analysis of genome-wide SNP data. Bioinformatics.

[36] Alexander DH, Novembre J, Lange K (2009). Fast model-based estimation of ancestry in unrelated individuals. Genome Res.

[37] Frichot E, François O (2015). LEA: an R package for landscape and ecological association studies. Methods Ecol Evol.

[38] Anderson EC, Thompson EA (2002). A model-based method for identifying species hybrids using multilocus genetic data. Genetics.

[39] Kamvar ZN, Tabima JF, Grunwald NJ (2014). Poppr: an R package for genetic analysis of populations with clonal, partially clonal, and/or sexual reproduction. PeerJ.

[40] Gutenkunst RN, Hernandez RD, Williamson SH, Bustamante CD (2009). Inferring the joint demographic history of multiple populations from multidimensional SNP frequency data. PLoS Genet.

[41] Portik DM, Leaché AD, Rivera D, Barej MF, Burger M, Hirschfeld M, Rödel M-O, Blackburn DC, Fujita MK (2017). Evaluating mechanisms of diversification in a Guineo-Congolian tropical forest frog using demographic model selection. Mol Ecol.

[42] Cornuet J-M, Pudlo P, Veyssier J, Dehne-Garcia A, Gautier M, Leblois R, Marin J-M, Estoup A (2014). DIYABC v2.0: a software to make approximate Bayesian computation inferences about population history using single nucleotide polymorphism, DNA sequence and microsatellite data. Bioinformatics..

[43] Corander J, Marttinen P, Sirén J, Tang J (2008). Enhanced Bayesian modelling in BAPS software for learning genetic structures of populations. BMC Bioinformatics.

[44] Villacorta-Rath C, Ilyushkina I, Strugnell JM, Green BS, Murphy NP, Doyle SR, Hall NE, Robinson AJ, Bell JJ (2016). Outlier SNPs enable food traceability of the southern rock lobster, *Jasus edwardsii*. Mar Biol.

[45] Farhadi A, Pichlmueller F, Yellapu B, Lavery S, Jeffs A. Genome-wide SNPs reveal fine-scale genetic structure in ornate spiny lobster *Panulirus ornatus* throughout indo-West Pacific Ocean. ICES J Mar Sci. 2022. 10.1093/icesjms/fsac130.

[46] Dang BT, Rahman MA, Tran SQ, Glenner H (2019). Genome-wide SNP analyses reveal population structure of *Portunus pelagicus* along Vietnam coastline. PLoS One.

[47] Lal MM, Southgate PC, Jerry DR, Bosserelle C, Zenger KR (2017). Swept away: ocean currents and seascape features influence genetic structure across the 18,000 km indo-Pacific distribution of a marine invertebrate, the black-lip pearl oyster *Pinctada margaritifera*. BMC Genomics.

[48] Singh SP, Groeneveld JC, Al-Marzouqi A, Willows-Munro S (2017). A molecular phylogeny of the spiny lobster *Panulirus homarus* highlights a separately evolving lineage from the Southwest Indian Ocean. PeerJ..

[49] Singh SP, Groeneveld JC, Hart-Davis MG, Backeberg BC, Willows-Munro S (2018). Seascape genetics of the spiny lobster *Panulirus homarus* in the Western Indian Ocean: understanding how oceanographic features shape the genetic structure of species with high larval dispersal potential. Ecol Evol.

[50] Torquato F, Range P, Ben-Hamadou R, Sigsgaard EE, Thomsen PF, Riera R, Berumen ML, Burt JA, Feary DA, Marshell A, D’Agostino D, DiBattista JD, Møller PR (2019). Consequences of marine barriers for genetic diversity of the coral-specialist yellowbar angelfish from the Northwestern Indian Ocean. Ecol Evol.

[51] Betzler C, Eberli GP, Lüdmann T, Reolid, J., Kroon, D., Reijmer, J. J. G., Swart. P. K., … Yao, Z. (2018). Refinement of Miocene Sea level and monsoon events from the sedimentary archive of the Maldives (Indian Ocean). Prog Earth Planet 5: 5.

[52] Sudheesh, V., Gupta, G., Reddy, Y., Bepari, K. F., Chari, N., Sherin, C. K., Shaju, S. … Vijayan, A. (2022). Oxygen minimum zone along the eastern Arabian Sea: intra-annual variation and dynamics based on ship-borne studies. Prog Oceanogr 201: 102742.

[53] Spalding MD, Fox HE, Allen GR, Davidson N, Ferdaña ZA, Finlayson M (2007). Marine ecoregions of the world: A bioregionalization of coastal and shelf areas. BioScience.

[54] Salas EM, Bernardi G, Berumen ML, Gaither MR, Rocha LA (2019). RADseq analyses reveal concordant Indian Ocean biogeographic and phylogeographic boundaries in the reef fish Dascyllus trimaculatus. R Soc Open Sci.

[55] Gaye B, Böll A, Segschneider J, Burdanowitz N, Emeis K-C, Ramaswamy V, Lahajnar N (2018). Glacial–interglacial changes and Holocene variations in Arabian Sea denitrification. Biogeosciences.

[56] Bialik OM, Frank M, Betzler C, Zammit R, Waldmann ND (2019). Two-step closure of the Miocene Indian Ocean gateway to the Mediterranean. Sci Rep.

[57] DiBattista JD, Berumen ML, Priest MA, De Brauwer M, Coker DJ, Sinclair-Taylor TH, Hay (2021). Environmental DNA reveals a multi-taxa biogeographic break across the Arabian Sea and sea of Oman. Environ DNA.

[58] DiBattista JD, Rocha LA, Hobbs J-PA, He S, Priest MA, Sinclair-Taylor TH, Bowen BW, Berumen ML (2015). When biogeographical provinces collide: hybridization of reef fishes at the crossroads of marine biogeographical provinces in the Arabian Sea. J Biogeogr.

[59] Michon L, Bachelery P, Lénat J-F, Di Muro A, Michon L (2016). The volcanism of the Comoros archipelago integrated at a regional scale. Active volcanoes of the Southwest Indian Ocean: piton de la Fournaise and Karthala, active volcanoes of the world, 978-3-642-31394-3.

[60] Teske P, von der Heyden S, McQuaid C, Barker N (2011). A review of marine phylogeography in southern Africa. S Afr J Sci.

[61] Palumbi, S. (1994). Genetic divergence, reproductive isolation, and marine speciation. Annu. Rev. Ecol. Evol. Syst . 25: 547–572. https://www.jstor.org/stable/2097324.

[62] Bagheri D, Farhadi A, Bargahi A, Nabipour I, Alavi Sharif S, Jeffs A (2020). Morphometric and genetic characterizations of blue swimming crab *Portunus segnis*, (Forskal, 1775) along the Iranian coasts of the Persian Gulf and Oman Sea. Reg Stud Mar Sci.

