# Supplementary material for: Genome-wide SNPs in the spiny lobster Panulirus homarus reveal a hybrid origin for its subspecies
Source: BMC Genomics. 2022 Nov 12;23:750. doi: 10.1186/s12864-022-08984-w (PMC9652991; doi:10.1186/s12864-022-08984-w)
Supplement: Supplementary file 1 — Additional file 1. The result of supportive analysis from of mtCR sequences and SNPs in this study. [file 12864_2022_8984_MOESM1_ESM.docx]

Supplementary file 1 for:

Fractions with too much friction; Genome-wide SNPs in the spiny lobster *Panulirus homarus* reveal a hybrid origin for its subspecies

Ahmad Farhadi^1, 2^

Andrew G Jeffs^2^

Shane D Lavery^2^

^1^Department of Natural Resources and Environmental Engineering, Shiraz University, Shiraz, Iran

^2^School of Biological Sciences, The University of Auckland, Auckland, New Zealand


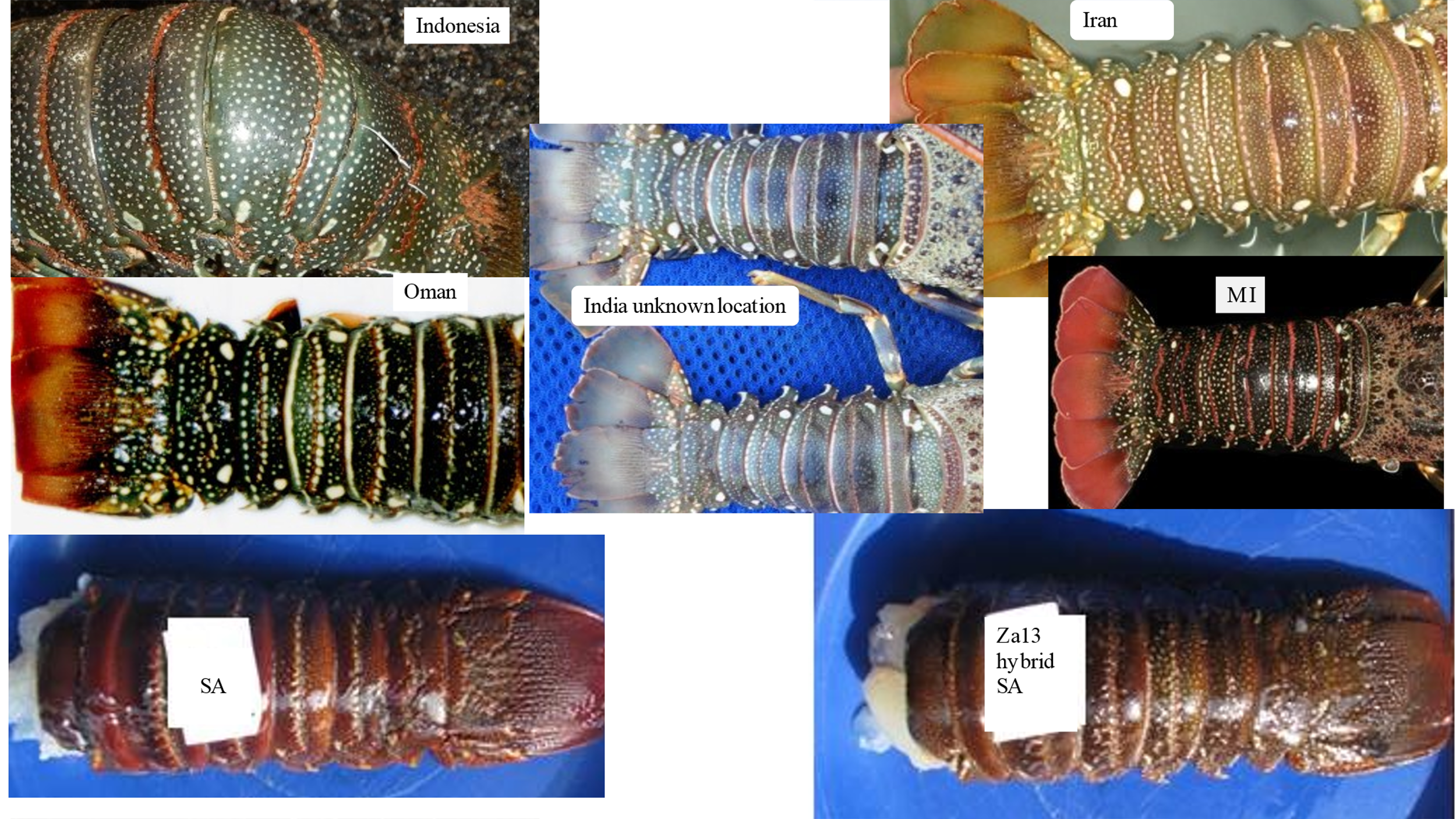


**Supplementary Figure S1**: main morphotypes of *Panulirus homarus* complex including *P. h. homarus* from Indonesia and India, *P*. *h. megasculpta* from Oman, *P. h. megasculpta* hybrid from Chabahar coast in Iran, *P. h. homarus* from Marquesas Island (MI), *P. h. rubellus* (SA) *P. h. rubellus* hybrid (Z13 hybrid SA) from South Africa east coast.


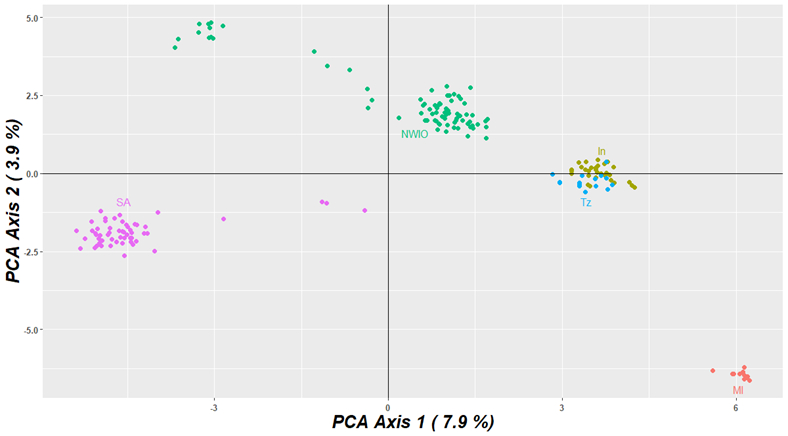


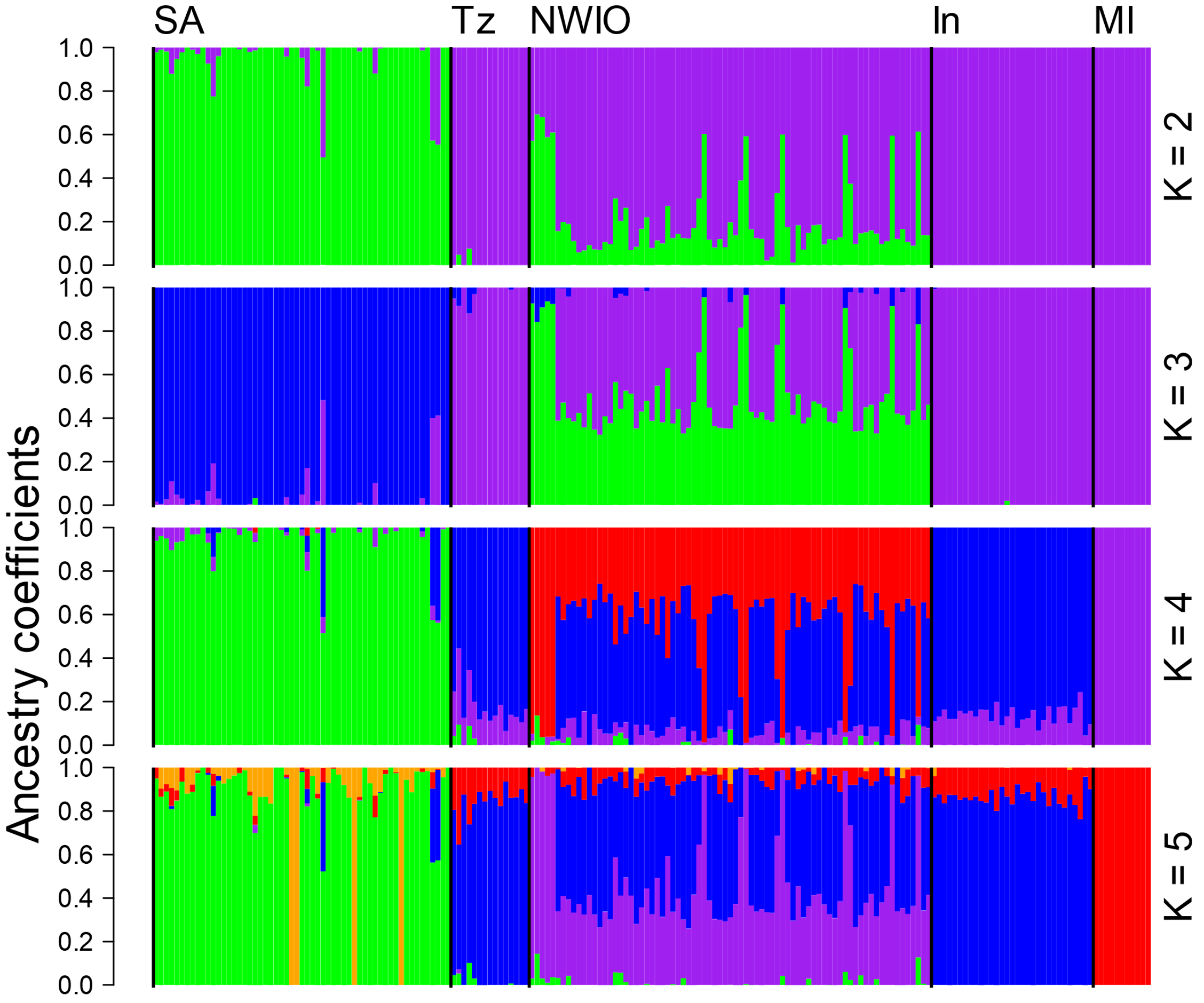


**Supplementary Figure S2:** PcoA plot of individual *P. homarus* and inference of individual admixture coefficient using sNMF K= 2-5 on *P. homarus* populations. Each vertical bar represents one individual.

**Supplementary Figure S3**: DAPC cluster of samples from NWIO and India representing the possible hybrid population between India and NWIO.

**Supplementary Figure S4**: Maximum likelihood phylogenetic trees constructed from SNP genotype divergences (on right) versus mtCR haplotype divergences (on left). Orange: SA, red: EA, Blue: NWIO, Green: C, purple: MI.

**Supplementary Figure S5**: Best-fitting demographic models selected using DIYABC. SC: secondary contact, Ne: population size change, IM2R: two immigration period. AM: ancient migration.


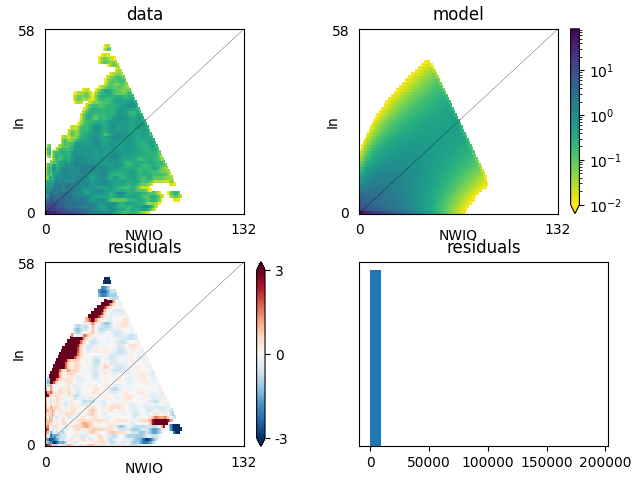

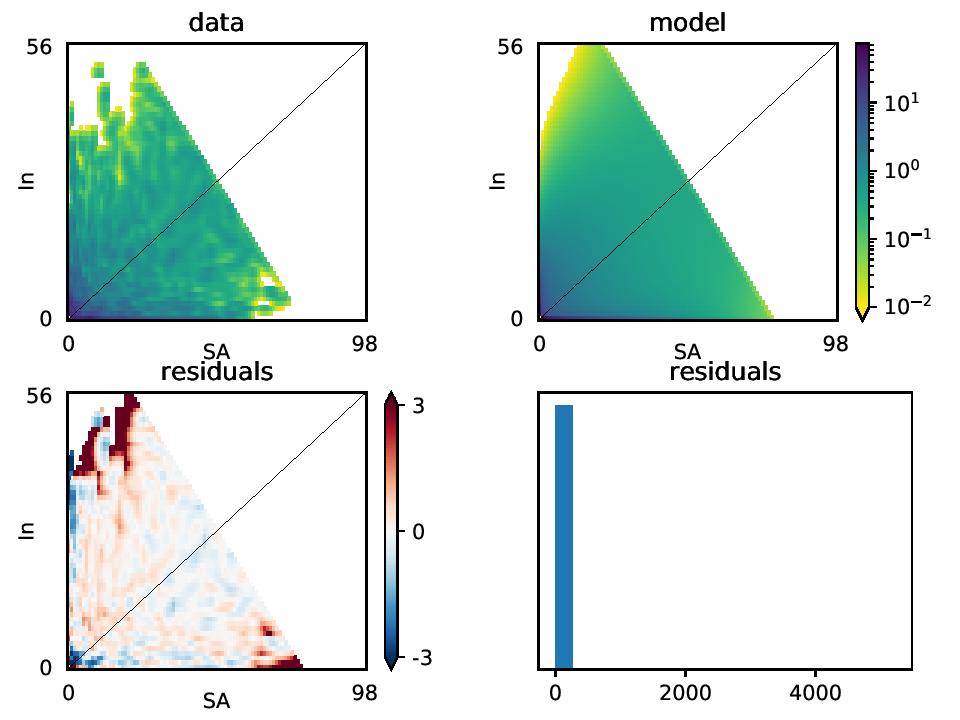

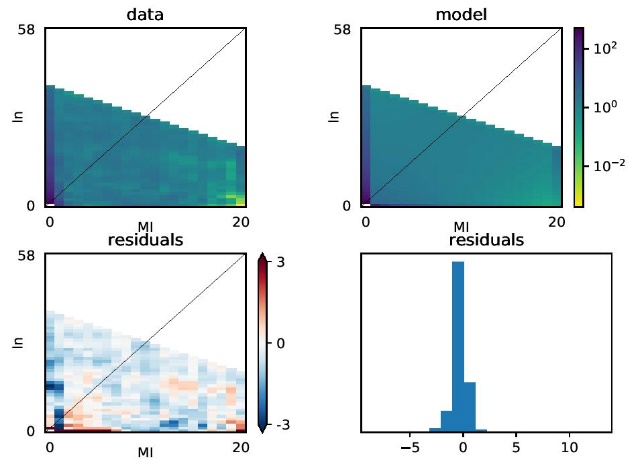

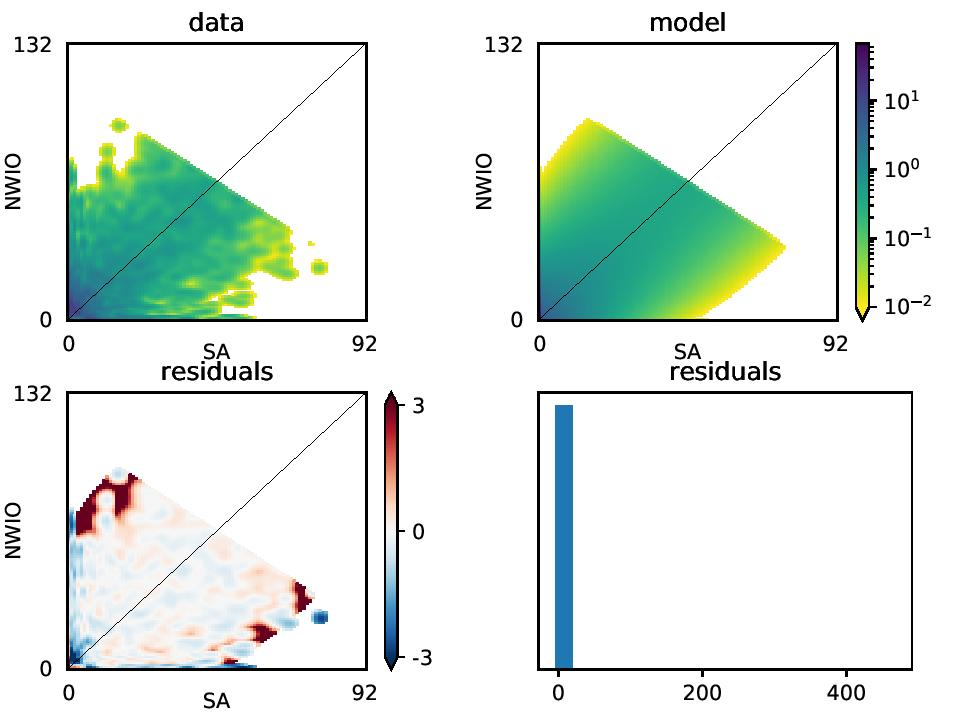


**Supplementary Figure S6**: Joint allele frequency spectrum (AFS) obtained from data (top right) and best fit model (top left) for linage pairs in *P. homarus* complex inferred from diffusion approximation method. a) In-MI, b) In-NWIO, c) In-SA d) NWIO-SA

**Supplementary Table S1**: Number of SNPs retained after each filtering step.

| Filtering step | SNP retained restricted filtering |
| --- | --- |
| Initial potential SNPs obtained from DArT | 103908 |
| Read depth above 5 filter | 82351 |
| Clustered SNPs filters | 34360 |
| Replication average | 33908 |
| Call rate | 12097 |
| MAF>0.02 | 4958 |
| Hamming distance filter | 3749 |
| Missingness ≥ 0.2 filter | 2807 |
| HWE filter &LD filter | 2020 |
| SNPs used for population genetic& demography analysis | 2020 |

**Supplementary Table S2**: Pairwise F_ST_ data from mtCR DNA sequences of *Panulirus homarus*

|  | SA | EA | NWIO | C | MI |
| --- | --- | --- | --- | --- | --- |
| SA | 0 |  |  |  |  |
| EA | 0.740 | 0 |  |  |  |
| NWIO | 0.757 | 0.119 | 0 |  |  |
| C | 0.716 | 0.0464 | 0.043 | 0 |  |
| MI | 0.758 | 0.716 | 0.627 | 0.528 | 0 |

All pairwise differences are significant at P<0.05

**Supplementary Table S3**: Five best-fitting models for each *P. homarus* population pair.

| Population pair | Model | Parameters | LogL | AIC | Θ |
| --- | --- | --- | --- | --- | --- |
| C-MI | S_asymig_size  Snomig_size  Snomig | 8  4  3 | -856  -989  -1161 | 1680  1812  1976 | 479  474  459 |
|  | Sanc_asymig  Sancasymig_size | 6  9 | -1385  -1315 | 2226  2240 | 215  111 |
| C-SA | Snomig_secont | 6 | -2025 | 4063 | 481 |
|  | Sasymig | 5 | -2180 | 4171 | 714 |
|  | Snomig-size-secont | 8 | -2213 | 4444 | 140 |
|  | Sanc_asymig  Snomig | 6  3 | -2834  -2303 | 5681  4493 | 165.1  623 |
| C-NWIO | Size_Ssize_secont | 11 | -2323 | 4468 | 107 |
|  | Ssymig | 4 | -2967.2 | 5121 | 815 |
|  | Ssymig_size | 7 | -2242 | 4498 | 886 |
|  | Snomig  No_divergence | 3  0 | -2421.6  -6212.8 | 4849  12435 | 519  421 |
| NWIO-SA | Snomig_size  Snomig | 6  3 | -3173  -3320 | 6365  6650 | 38  58 |
|  | Sancasymig_size  Ssecont_asymig_size  Ssecont_symig_size | 8  8  7 | -3389  -3505  -3850 | 6785  7026  7840 | 549  86  43 |

Abbreviations: C: central (*P. homarus*), MI: Marquesas Islands, NWIO: Northwest Indian Ocean (*P*. *h. megasculpta*), SA: South Africa (*P. h. rubellus*) LogL: best maximum likelihood estimates for 100 runs, AIC: Akaike weight of model, Θ: theta parameter for the ancestral population before split (θ = 4Nrefµ), with N_ref_ being the effective size of the ancestral population, and μ the per-site mutation rate per generation.

Abbreviations of model names; S: split, symig: symmetric migration, asymig: asymmetric migration, secont (secondary contact with assumption of asymmetric gene flow), amig: ancient migration, nomig: no migration. Size: historical population size change.

**Supplementary Table S4**: Historical demography indices from mtCR and SNP data of lineages in *Panulirus* *homarus* evolution.

| Lineage | Tajima’s D SNP scaled | Tajima’s D mtCR (P value) | Theta pi mtCR | Fu’s F_S_ (P value) |
| --- | --- | --- | --- | --- |
| *P. homarus* C | -0.180 | -1.021 (0.154) | 25.34 | -14.354 (0.001) |
| *P. h. rubellus* SA | -0.186 | -1.430 (0.057) | 35.72 | -24.163 (0.0000) |
| *P. homarus* NWIO | -0.180 | -1.308 (0.062) | 35.84 | -24.060 (0.0000) |
| Marquesas Island | -0.092 | 0.959 (0.872) | 12.26 | -3.51 (0.040) |
| All (mean) | -0.159 | -0.787 (0.251) | 25.38 | -13.823 (0.010) |
